# Trimethylamine-*N*-oxide is an independent risk factor for hospitalization events in patients receiving maintenance hemodialysis

**DOI:** 10.1080/0886022X.2020.1781170

**Published:** 2020-06-24

**Authors:** Yin Zheng, Zihui Tang, Li You, Yuanhao Wu, Junfeng Liu, Jun Xue

**Affiliations:** a Department of Nephrology, Huashan Hospital, Fudan University, Shanghai, China; b Department of Integrative Medicine, Huashan Hospital, Fudan University, Shanghai, China

**Keywords:** Chronic kidney disease, hemodialysis, hospitalization events, trimethylamine, trimethylamine-*N*-oxide

## Abstract

**Background:**

Hospitalization is a significant outcome measurement for maintenance hemodialysis pantients. Trimethylamine-*N*-oxide (TMAO), created by gut microflora from dietary l-carnitine and choline, cleared by the kidney, has been implicated in the causation of cardiovascular diseases in patients with chronic kidney disease. However, whether it associates with hospitalization risk for these patients is unclear.

**Methods:**

In this study, 69 patients undergoing outpatient dialysis were enrolled. Enzyme-linked immunosorbent assay was used to quantitate the baseline plasma TMAO levels in patients. The patients were divided into a high TMAO level group (TMAO ≥ 15 μmol/L) and a low TMAO level group (TMAO < 15 μmol/L). During the 1-year follow-up, 1-year dialysis-related data and all-cause hospitalization events were recorded.

**Results:**

The incidence of hospitalization events was significantly higher in the high TMAO level group than in the low TMAO level group (91 per 100 patient-year vs. 32 per 100 patient-year). The Kaplain–Meier survaial analysis showed that the incidence of hospitalization events in the high TMAO level group was significantly higher than that in the low TMAO level group (log-rank *p* = 0.0004). After adjustment age, sex, CK-MB and albumin, the results of multivariate Cox proportional hazard analysis showed that high TMAO level was an independent risk factor for hospitalization in maintenance hemodialysis patients.

**Conclusion:**

TMAO is an independent risk factor for hospitalization events in patients receiving maintenance hemodialysis. It may be a new therapeutic target for improving the outcomes of these patients.

## Introduction

Chronic kidney disease (CKD) is a global public health concern and is a particularly serious problem in developing countries [1]. Compared with the population with normal renal function, patients with CKD have many factors that significantly increase their risks for hospitalization events [2]. There are many common causes of hospitalization in patients with CKD. In addition to the common cardiovascular diseases, lung infection, sepsis, bile duct diseases, the arteriovenous fistula disorders, and etc., are all closely associated with hospitalization events in patients with CKD [3–5]. However, the risk factors that increase hospitalization events in these patients have always been controversial. Although cardiovascular diseases are the most common causes of hospitalization in patients with CKD, it has been reported that conventional risk factors such as diabetes, hypertension, and hyperlipidemia do not accelerate vascular stenosis in these patients [6]. The results of previous studies showed that there are other special risk factors in this population [7].

Recently, some studies have shown that protein-binding toxins synthesized by the gut microbiota, such as trimethylamine-*N*-oxide (TMAO), are intimately associated with the cardiovascular disease outcomes in patients with CKD [8]. TMAO is an enterogenous micromolecular toxin that is mainly synthesized by the gut microflora, and its precursor is trimethylamine (TMA) [9].The gut microbiota uses TMA lyase to degrade biliary lipids, lecithin, carnitine, and other nutrients to produce TMA, which enters the liver through the portal circulation. In the liver, flavin-containing mono-oxygenase 3 (FMO3) converts TMA to TMAO [10,11]. TMAO in plasma can regulate lipid and glucose homeostasis, and promote the development of chronic diseases such as atherosclerosis, diabetes, and CKD [12,13]. As TMAO is mainly excreted through urine, the serum TMAO levels of patients with CKD are several times higher than those of healthy people. Recently, some studies found that elevated TMAO levels significantly increase the risk of cardiovascular diseases in patients with CKD [14,15]. However, in patients who undergo hemodialysis, whether TMAO elevation is associated with cardiovascular disease remains debatable [16,17]. Therefore, there are reasons to believe that the effects of TMAO on patients undergoing dialysis are multifaceted.

Hospitalization is a significant outcome measurement because it provides insight into the morbidity and the cost of treatment among dialysis patients [18]. Currently, there is limited research on the risk factors for hospitalization events in patients undergoing hemodialysis. In addition, it is not clear whether TMAO is associated with hospitalization events in these patients. Therefore, we measured laboratory markers such as TMAO in patients undergoing maintenance hemodialysis in our center and conducted follow-up evaluations of all hospitalization events in these patients over one year to examine the relevant risk factors for hospitalization events in maintenance hemodialysis patients.

## Materials and methods

### General condition

A total of 69 patients undergoing outpatient maintenance hemodialysis in the hemodialysis center of Fudan University Affiliated Huashan Hospital North Hospital were included in this study. We excluded the subjects who took broad-spectrum antibiotics within one week and with malignant tumor whose expecting life were less than one year. All patients were received three times hemodialysis a week. Each session is four hours. The patients were followed up from 1 June 2017 to 31 May 2018 for all-cause hospitalization events. The study protocol was approved by the Ethics Committees of Huashan Hospital (approval no.KY2016-394).

### Data collection

Demographic and dialysis-related data were collected: age, sex, body mass index, vascular access, dialysis vintage, blood pressure before and during dialysis, comorbidities, and the increase in body weight during dialysis (body weight before dialysis – body weight after previous dialysis).

Venous blood was collected from the 69 patients in fasting and resting status before mid-week dialysis following enrollment. The blood samples were centrifuged appropriately to obtain plasma samples, which were stored at −80 °C. Laboratory indexes were detected using the blood samples, including blood cell count, hemoglobin (Hb), albumin (Alb), prealbumin, creatine kinase isoenzyme (CK-MB), troponin I (TnT), N-Terminal pro-brain natriuretic peptide (NT-proBNP), β2 microglobulin, blood urea nitrogen (BUN), creatinine (Scr), high sensitivity C-reactive protein (hs-CRP), transferrin saturation (TSAT), intact parathyroid hormone (iPTH), blood calcium (Ca), and blood phosphorus (P), total cholesterol (CHO), low-density lipoprotein cholesterol (LDL), etc. A 5 mL volume was collected from the median cubital vein of the patients into an EDTA tube, centrifuged at 12,000 × *g* at 4 °C for 15 min, and then the supernatant was collected. Enzyme-linked immunosorbent assay (TMAO ELISA kit, MIBio, Shanghai, China) was employed to measure plasma TMAO levels.

### Outcomes

All-cause hospitalizations were documented. Cardiovascular hospitalizations were defined as hospitalizations for myocardial infarction, ischemic heart disease, heart failure, arrhythmias, stroke and peripheral vascular event. The hospitalizations for fistular dysfuntion were documented. The events of autologous or graft internal fistular dysfunction included 1) the reduction of flow rate (flow rate of graft internal fistula <600 mL/min and of autologous internal fistula <500 mL/min); 2) thrombosis; 3) occlusion; 4) infection. The hospitalization rates defined as the number of admissions divided by 100 person years (PY) of follow-up.

### Statistical methods

Continuous variables were expressed as mean ± standard deviation or median (interquartile range), and categorical variables are expressed as number and percentage. Student’s *t*-test or Mann–Whitney *U* test (non-normal distribution) was used to compare the differences in continuous variables, and the Chi-square test or Fisher’s exact test was used to compare categorical variables. The patients were divided into the high TMAO group (TMAO ≥ 15 μmol/L) and low TMAO group (TMAO < 15 μmol/L) according to the cutoff point. The cutoff point of serum TMAO was calculated by obtaining the highest Youden index. The cumulative incidence of hospitalization was described using the Kaplan–Meier method and were statistically tested with the log-rank test. Cox proportional hazard model was used to analysis the impact of baseline characteristics, laboratory indexes and TMAO on time to hospitalization. For arteriovenous fistula disfunction and cardiovascular event, Cox proportional hazard analysis was not perfomed because of the very small number of these events. A difference of *p* < 0.05 was considered to be statistically significant. The SPSS 21.0 statistical software (IBM Corp, Armonk, NY) was used for data processing and analysis .

## Results

### Comparison of general information of the two groups of patients

A total of 69 patients undergoing dialysis were included, comprising 36 (52.2%) men and 33 (47.8%) women. The mean age was 61.28 ± 11.99 years, and the median dialysis vintages was 37 (26.5–81) months. The causes of ESRD included chronic glomerulonephritis (*n* = 27, 39.1%), hypertensive nephropathy (*n* = 19, 27.5%), polycystic kidney disease (*n* = 7,10.1%), diabetic nephropathy (*n* = 6, 8.7%), obstructive nephropathy (*n* = 5, 7.2%), and other causes (*n* = 5, 7.2%). Baseline characteristics were well balanced between the groups. There were no statistical differences in the markers of myocardial injury, hs-CRP, hemoglobin, β2 microglobulin, prealbumin, albumin, predialysis Scr, predialysis BUN, calcium, phosphorus, and iPTH levels, iron metabolism, and lipids between the two groups (Table 1).

**Table 1.** Demographic and clinical characteristics of hemodialysis (HD) patients according to different level of trimethylamine oxide.

Variables	Total(*n* = 69)	TMAO < 15μmol/L(*n* = 47)	TMA*O* ≥ 15μmol/L(*n* = 22)	*p* Value
Age (years)	61.28 ± 11.99	60.28 ± 11.13	63.41 ± 13.68	0.316
Male, n (%)	36 (52.2%)	26 (55.3%)	10 (45.5%)	0.093
Dialysis vintages (months)	37 (26.5–81.0)	36.0 (25.0–76.0)	50.0 (30.8–86.8）	0.282
Body mass index, kg/m^2^	21.67 ± 3.21	21.52 ± 3.04	21.36 ± 3.61	0.683
Vascular access (AVF %)	57 (82.6%)	39 (83.0%)	18 (81.9%)	1.000
Diabetes, n (%)	9 (13.0%)	7 (14.9%)	2 (9.0%)	0.777
Hypertension, n (%)	61 (88.40%)	41 (87.2%)	20 (90.9%)	0.967
Predialysis SBP	141.98 ± 13.64	141.93 ± 13.56	142.08 ± 14.12	0.967
Predialysis DBP	80.12 ± 9.07	80.65 ± 9.19	78.97 ± 8.91	0.477
IDWG (kg)	2.02 ± 0.88	2.04 ± 0.93	1.97 ± 0.79	0.780
IDH (%)	0.025 (0–0.147)	0.025 (0–0.086）	0.027 (0–0.195)	0.683
NT-proBNP (pg/ml)	2488 (1429–4935)	2075 (1225–4672）	2834 (1557–5146)	0.421
CK-MB (ng/ml)	1.63 (1.26–2.51)	1.55 (1.25–2.1)	1.92 (1.24–3.64）	0.221
TnT (ng/ml)	0.038 (0.027–0.068)	0.038 (0.028–0.065）	0.041 (0.025–0.083)	0.832
hsCRP (mg/L)	3.2 (0.92–5.49)	2.26 (0.78–4.75)	4.07 (1.34–6.05)	0.200
RBC (*10^12^/L)	3.53 (3.25–3.81)	3.58 (3.28–3.82)	3.41 (3.21–3.81)	0.571
Hb (g/L)	106 (95.5–115.5)	106.0 (98–116)	102.5 (94–114.3)	0.723
β_2_MG (mg/L)	17.22 (15.02–18.67)	17.05 (14.27–18.41)	17.90 (15.80–19.15)	0.135
Prealbumin (g/L)	334.45 ± 75.40	333.64 ± 73.93	336.18 ± 80.19	0.897
Alb (g/L)	41.80 ± 4.56	41.80 ± 3.44	42.10 ± 6.37	0.400
Predialysis BUN (mmol/L)	27.97 ± 6.02	28.62 ± 6.30	26.60 ± 5.26	0.200
Predialysis Scr (μmol/L)	1014.19 ± 244.33	1045.70 ± 244.29	947.0 ± 235.88	0.119
Ca (mmol/L)	2.31 ± 0.19	2.29 ± 0.20	2.36 ± 0.17	0.125
P (mmol/L)	1.98 (1.74–2.40)	1.98 (1.68–2.49）	1.95 (1.78–2.27)	0.974
iPTH (ng/L)	353.2 (173.6–616.1)	395.5 (167–594.6)	352.7 (176.58–789.28)	0.616
Ferrintin (μg/L)	264.1 (143.7–443.3)	264.1 (124.6–474.2)	256.1 (186.88–306.5)	0.757
TSAT %	27.0 (22.0–36.5)	29.0 (23.0–38.0)	24 (20.0–30.75)	0.078
CHO (mmol/L)	4.10 (3.56–4.92)	4.92 ± 4.53	4.37 ± 0.99	0.503
LDL (mmol/L)	2.26 (1.76–2.94)	2.19 (1.71–2.5)	2.81(1.87–3.42)	0.093

TMAO: trimethylamine-*N*-oxide; AVF: arteriovenous fistula；SBP: systolic blood pressure; DBP: diastolic blood pressure; IDWG: interdialytic weight gain; IDH (%): incidence of interdialytic hypotension; NT-proBNP: N-Terminal pro-brain natriuretic peptide; CK-MB: creatine kinase isoenzyme; TnT: troponin I; hsCRP: high sensitivity C-reactive protein; iPTH: immunoreactive parathyroid hormone; RBC: red blood cell; Hb: hemoglobin; β2MG:β2 microglobulin; Alb: albumin; BUN: blood urea nitrogen; Scr: creatinine; Ca: calcium; P: phosphorus; TSAT: transferrin saturation; CHO: cholesterol; LDL: low-density lipoprotein.

### Relationship between plasma TMAO levels and hospitalization events

There were total 35 hospitalization events in both group during one year follow-up, 20 events for the high TMAO level group and fifteen events for the low TMAO level group. The total hospitalization rates (per 100 patient-years) were 91 in the high TMAO group and 32 in the low TMAO group. The causes of hospitalization in the high TMAO group included the incidents of arteriovenous fistula disfunction events (*n* = 9, 45%), cardiovascular disease events (*n* = 4, 20%), parathyroidectomy (*n* = 3, 15%), pneumonia (*n* = 1, 5%), urinary tract infection (*n* = 1, 5%), lung cancer (*n* = 1, 5%) and depression (*n* = 1, 5%). The causes of hospitalization in the low TMAO group included the incidents of arteriovenous fistula disfunction events (*n* = 4, 27%), cardiovascular disease events (*n* = 3, 20%), pneumonia (*n* = 2, 13%), urinary tract infection (*n* = 2, 13%), parathyroidectomy (*n* = 1, 7%), gastric ulcer (*n* = 1, 7%), uterus cancer (*n* = 1, 7%) and car accident (*n* = 1, 7%).

### TMAO level is an independent risk factor for hospitalization event

**Figure 1.**
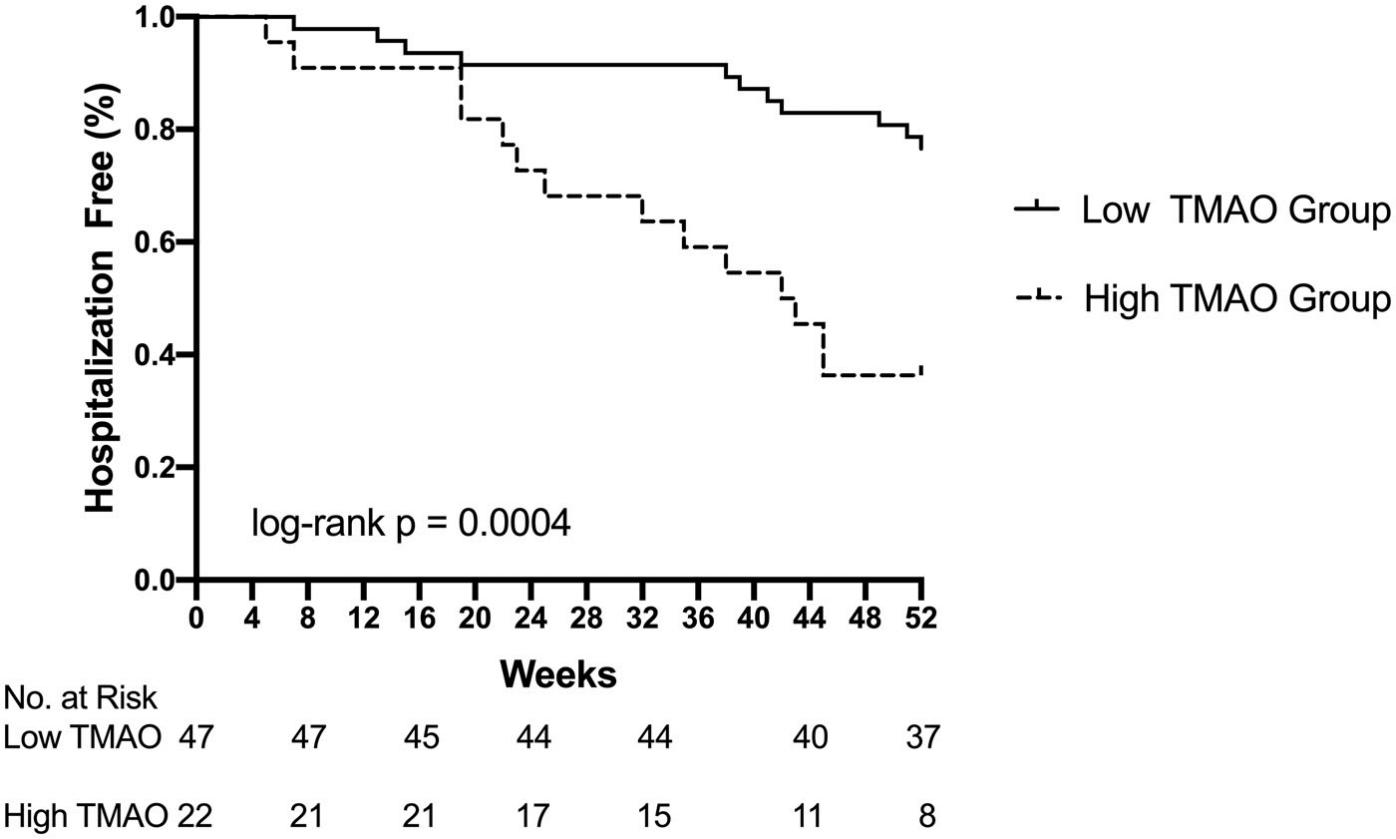
Kaplan–Meier estimates for hospitalization events by the cutoff values of TMAO. Maintenance hemodialysis patients with serum TMAO levels (≥15 μmol/L) had more hospitalization events. (log-rank *p* = 0.0004).

The Kaplain–Meier survaial analysis showed that the incidence of hospitalizations in the high TMAO level group was significantly higher than that in the low TMAO level group (log-rank *p* = 0.0004) (Figure 1). Univariate Cox proportional hazard analysis showed that only high TMAO level (≥15μmol/L) had significantly impact on hospitalization (unadjusted HR: 2.303; 95%CI: 1.031–5.144; *p* = 0.042). (Table 2) After adjustment for age and sex, high TMAO level (≥15μmol/L) was the only parameter entered into the multivariate analysis. (adjusted HR: 2.295; 95%CI:1.008–5.225; *p* = 0.048). On multivariate Cox proportional hazard analysis included TMAO, CK-MB, albumin, high TMAO level was consistently retained as indepent risk factors for hospitalization (Table 3).

**Table 2.** Univariate Cox regression analysis for factors predicting hospitalization events.

Covariates	Unit of increase	HR (95% CI)	*p* Value
Age	1yr	1.034 (0.991–1.079)	0.124
Sex	Male	1.062 (0.465–2.426)	0.887
Dialysis vintage	1 mo	0.998 (0.991–1.006)	0.628
BMI (kg/m^2^)	<20	1.000	0.766
	20 ≤ BMI<24	1.095 (0.398–3.013)	0.861
	>24	0.779 (0.270–2.245)	0.643
Vascular access	AVF versus CVC	2.102 (0.871–5.071)	0.098
Diabetes	Yes versus no	2.491 (0.988–6.279)	0.053
Hypertension	Yes versus no	3.267 (0.441–24.199)	0.247
IDWG	1kg	0.819 (0.515–1.303)	0.400
IDH	1%	0.182 (0.009–3.748)	0.270
NT-proBNP (pg/ml)	<1000	1.000	0.437
	1000 ≤ NT-proBNP <10000	0.241 (0.025–2.322)	0.218
	≥10000	0.570 (0.169–1.918)	0.364
CK-MB	1 ng/ml	1.163 (0.982–1.378)	0.080
hsCRP	1 mg/L	1.013 (0.998–1.029)	0.085
Hb	1g/L	0.999 (0.973–1.025)	0.921
Albumin	1 g/L	1.001 (0.918–1.090)	0.988
Predialysis creatinine	1μmol/L	0.999(0.998–1.001)	0.569
iPTH	1 ng/L	1.000 (0.999–1.001)	0.852
P	1mmol/L	1.695 (0.980–2.934)	0.059
Ca	1mmol/L	0.376 (0.044–3.206)	0.371
LDL	1mmol/L	1.062 (0.879–1.283)	0.534
TMAO(entered as categorical variable)	High versus low	2.303 (1.031–5.144)	0.042

BMI: body mass index; SBP systolic blood pressure; DBP: diastolic blood pressure; AVF: arteriovenous fistula; CVC: central venous catheter; IDWG: interdialytic weight gain; IDH (%): incidence of interdialytic hypotension; NT-proBNP: N-Terminal pro-brain natriuretic peptide; CK-MB: creatine kinase isoenzyme; hsCRP: high sensitivity C-reactive protein; iPTH: immunoreactive parathyroid hormone; Hb: hemoglobin; Alb: albumin; Scr:creatinine; Ca: calcium; P: phosphorus; LDL: low-density lipoprotein; TMAO: trimethylamine-*N*-oxide; HR: hazard ratio.

**Table 3.** Multivariate Cox regression analysis for factors predicting hospitalization events.

Covariates	Model 1	*p* Value	Model 2	*p* Value	Model 3	*p* Value
	HR (95% CI)		HR (95% CI)		HR (95% CI)	
Age	1.025 (0.981–1.072)	0.267	1.031 (0.989–1.075)	0.152		
Male	1.393 (0.660–2.941)	0.384				
TMAO (entered as categorical variable)	2.295 (1.008–5.225)	0.048	2.37 (1.062–5.293)	0.035	2.335 (1.040–5.242)	0.040
CK-MB			1.216 (0.995–1.486)	0.056		
Albumin					0.990 (0.918–1.067)	0.787

CK-MB: creatine kinase isoenzyme; HR: hazard ratio.

## Discussion

Recent studies found that the enterogenous toxin TMAO is closely associated with the occurrence and development of cardiovascular events in patients with uremia [19–22]. In recent years, TMAO was found to be possibly cytotoxic to mammals. Experimental data suggest that TMAO may directly enhance atherogenesis and contribute to cardiovascular events *via* dysregulation of lipid handling and macrophage function as well as directly causing vascular inflammation and platelet activation leading to thrombosis. Elevated TMAO levels may lead to the occurrence and development of certain chronic diseases such as atherosclerosis, hypertension, and cardiac insufficiency. Tang et al. [23] further proved that TMAO is closely associated with chronic cardiovascular diseases. The higher the blood TMAO level, the greater the incidence of myocardial infarction, stroke, or mortality. Although TMAO has been proved to be closely associated with cardiovascular disease outcomes of patients with CKD not requiring dialysis, this result has always been controversial for patients undergoing hemodialysis. In Stubbs et al. [17] study, the researchers measured the TMAO levels in 1243 patients from the EVOLVE clinical trial [24], and found that TMAO was not associated with the composite cardiovascular disease mortality rate, myocardial infarction, peripheral vasculopathy, and other diseases. This shows that the effects of TMAO in humans are not limited to cardiovascular diseases. Therefore, Chhibber-Goel et al. [25] considered TMAO to be a new marker for human health, as elevated TMAO is associated with various diseases. In fact, elevated TMAO levels were also found to be associated with diabetes [26], pneumonia [27], tumor formation [28], and other diseases, in addition to cardiovascular disease. Therefore, elevated TMAO will significantly increase all-cause hospitalization events in patients. To our knowledge, this study is the first to examine the correlation between TMAO and hospitalization events in patients undergoing dialysis.

In this study, we enrolled 69 patients undergoing outpatient hemodialysis and divided them into the high and low TMAO level groups. We conducted follow-up evaluations of hospitalization events in these patients over one year. The result showed that the maintenance hemodialysis patients with higher TMAO level had higher risk of hospitalization. The arteriovenous fistula dysfunction and cardiovascular disease were the top two causes of hospitalization for both of groups. For the short follow-up, the number of cardiovascular events was small and we did not observe the differences of cardiovascular events between two groups. However, we observed that the dialysis patients with high TMAO were more likely to suffer from vascular access dysfunction than those with low TMAO in one-year follow-up, although they had no differences in the complication of hypertension and diabetic mellitus, level of incidence of interdialytic hypotension, calcium, phosphorus and iPTH, and low-density lipoprotein. On one hand, it demonstrated TMAO might take part into the vascular access sclerosis or thrombosis. On the other hand, because TMAO is both an osmolyte and a protein stabilizer, it has multiple effects on the body and tends to cause various diseases in patients, which increases hospitalization events [29]. Our study in patients undergoing hemodialysis also confirmed this point. Further study is needed to evaluate the risk of increased arteriovenous fistula events in chronic hemodialysis patients with high TMAO levels.

The multivariate Cox regression analysis showed that the TMAO level is an independent risk factor for hospitalization in patients undergoing hemodialysis. TMAO is a potential marker for the clinical assessment of outcomes in these patients. At present, some researchers believe that TMAO is a new therapeutic target for improving the outcomes of patients with uremia [19].

This study has some limitations. First, the sample size is small. The second limitation is the short follow-up period, which translates to few cardiovascular events. The third limitation is the lack of dietary data of the patients. Whether the timing of blood sample collection and the type of food intake have any effects on the quantitation of TMAO levels remain unclear.

In summary, our study has proven that TMAO level is an independent risk factor for all-cause hospitalization events in patients undergoing maintenance hemodialysis. Further studies are needed to determine whether decreasing TMAO levels will slow the progression of vascular disease and improve outcome in these patients.
